# Environmental Exposure Assessment of Pesticides in Farmworker Homes

**DOI:** 10.1289/ehp.8530

**Published:** 2006-02-16

**Authors:** Jane A. Hoppin, John L. Adgate, Monty Eberhart, Marcia Nishioka, P. Barry Ryan

**Affiliations:** 1 Epidemiology Branch, National Institute of Environmental Health Sciences, National Institutes of Health, Department of Health and Human Services, Research Triangle Park, North Carolina, USA; 2 School of Public Health, University of Minnesota, Minneapolis, Minnesota, USA; 3 Bayer CropScience, Research Triangle Park, North Carolina, USA; 4 Battelle Memorial Institute, Columbus, Ohio, USA; 5 Emory University, Atlanta, Georgia, USA

**Keywords:** dust sampling, farmworkers, pesticides

## Abstract

Farmworkers and their families are exposed to pesticides both at work and in their homes. Environmental exposure assessment provides a means to evaluate pesticides in the environment and human contact with these chemicals through identification of sources and routes of exposure. To date, a variety of methods have been used to assess pesticide exposure among farmworker families, mostly focusing on dust and handwipe samples. While many of the methods are similar, differences in the collection, chemical analysis, and statistical analysis, can limit the comparability of results from farm-worker studies. This mini-monograph discusses the strategies used to assess pesticide exposures, presents limitations in the available data for farmworkers, and suggests research needs for future studies of pesticide exposure among farmworker families.

Farmworkers are exposed to pesticides in both agricultural and residential settings. Although exposure during occupational activities likely results in their highest exposures, these events are episodic. Farmworkers are also subject to long-term, low-level exposures through drinking water and food as well as to pesticides that infiltrate or are tracked into their residences. Research has shown that these “paraoccupational” exposures may be more substantial than and occur more frequently than occupational exposures (Fenske 1997). Indeed, paraoccupational or carry-home exposures may be important sources of pesticide exposure for farmworker families.

Exposure assessment provides a key piece in the picture that describes farmworker exposure to pesticides in the field and at home. The reasons for conducting exposure assessments for farmworkers include exposure surveillance and characterization, intervention, and epidemiologic studies. In this article we discuss environmental exposure assessment techniques, with a focus on methods available for use in residences. Research in this area is multidisciplinary, and confusion often exists regarding the meaning of important terms such as “exposure” and “dose,” so we begin this overview by defining some critical terms. We then discuss the difference between exposure measurement and assessment, evaluate existing sampling and analysis techniques for characterizing pesticide concentrations in environmental media, and discuss important study design issues that need to be considered to facilitate comparisons across studies. We close by summarizing exposure assessment research needs for studies of farmworker families.

## Important Terms in Exposure Assessment

**Figure 1:**
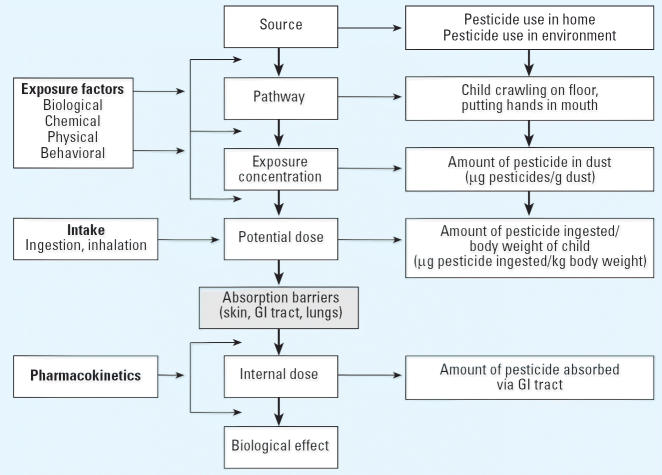
Exposure assessment paradigm. GI, gastrointestinal.

Exposure is a deceptively simple concept, defined as contact at a body boundary between a person and an environmental stressor (biological, chemical, or physical) over time (Ott 1985; Sexton et al. 1995; Zartarian et al. 2005) (Figure 1). This simple definition masks the fact that a quantitative exposure analysis requires collection and analysis of multiple parameters such as concentration and duration of exposure as well as exposure factors that affect contact rates and, therefore, determine the magnitude of exposure. A description of exposure for a particular route (i.e., inhalation, ingestion, or dermal absorption) must include at least the following two related attributes: concentration of the pesticide in the carrier medium and the duration of contact. Therefore, exposure to pesticides in the environment requires not only the presence of the pesticide, but also that an individual come in contact with the pesticide at a specific time in a specific place. If there is no possibility of contact, there is no exposure. Dose, as mentioned in the introductory article to this mini-monograph (Arcury et al. 2006), refers to levels measured within a biological boundary. Biomonitoring is the subject of an accompanying article of this series (Barr et al. 2006) and will not be discussed in detail here.

Concentration is the amount of pesticide measured in a mass or volume of an environmental medium. In residential exposure assessment, we are concerned with contact with residues found in water, food, dust, and air in and around the home. Most often, exposure concentration is expressed in units of mass pesticide/mass matrix (e.g., micrograms of pesticide per kilogram of dust). In some cases such as surface residue sampling, pesticide loading is the exposure metric used. Loading measures the amount of a chemical found over a unit area (e.g., micrograms of pesticide per square centimeter) or per unit measured (e.g., microgram per child’s hand).

Lastly, frequency and duration of exposure are key elements of pesticide exposure assessment because these variables are used to determine the cumulative dose over time. Frequency describes the number of contacts over a period of time (e.g., contact rate), and duration describes the lengths of these contacts. Exposures to pesticides typically vary over time with specific events such as applications indoors or to nearby fields appearing as spikes in an individual’s exposure profile over time above an individual’s background rate of exposure. Thus, estimating an average exposure for an individual may underestimate the impact of peak exposure events.

## Pesticide Exposure Assessment

The physical and chemical properties of pesticides will determine where and when they may be detected in the environment (Ware 1994). Temporal variability in exposure is influenced by application intensity, frequency, method of application, behavioral characteristics of the applicator (e.g., use of personal protective equipment, hand washing), as well as the physical, chemical, and biological properties of the pesticide formulation. The frequency and duration of exposure measurement are important design considerations for characterizing the temporal patterns of exposure to pesticides, and they are often determined by the persistence of pesticides and our ability to measure them in the environment. The persistent pesticide DDT (dichlorodiphenyltrichloroethane) as well as its primary metabolite and degradation product DDE (dichlorodiphenyldichloroethylene), for example, are still measurable in house dust in the United States more than 30 years after its use was banned (Lewis et al. 1995; Whitmore et al. 1994). The most widely used pesticides today, organophosphates, carbamates, and pyrethroids, are less persistent than older formulations. Therefore, it is important to measure exposures near the time of application because these compounds eventually degrade over time. Although data are limited, it is likely that pesticides in common use today degrade less quickly in indoor environments than they would outdoors because they are protected from sunlight, rain, temperature extremes, microbial action, and other degradation processes (Butte and Heinzow 2002; Lewis et al. 2001; Simcox et al. 1995). Therefore, pesticides in homes may represent a longer-term potential source of exposure, with relatively less temporal variability than outdoor applications.

## Exposure Assessment Approaches

In the most general sense, the quantitative estimation of chemical pesticide exposure can be approached in any of the following three ways: personal measurements, scenario-based assessment, and reconstructive analyses of biological measurements (Barr et al. 2006; Sexton et al. 1995). Personal measurements document exposures as they occur by measuring the pesticide concentration at the point of contact between the person and the environmental medium where the pesticide exists. Examples include use of pumps and filters to measure airborne concentrations near the breathing zone, duplicate diet food samples to measure dietary levels, or skin patch samples to measure dermal concentrations. The major strength of the personal measurement approach is that exposure is measured directly during the monitoring period, typically on the order of minutes, hours, or, at most, days. The problems with personal measurements are that they are costly and time consuming, they can be burdensome for the study participants, and suitable monitoring devices are not available for all pesticides and pathways of interest. Because these problems are exacerbated in the case of children, personal monitoring has rarely been attempted in this subpopulation (Weaver et al. 1998), thereby limiting its use in assessments involving the children of farmworkers.

Biological measures of exposure may be used to “reconstruct” dose from body burden measurements if information or assumptions about rates of intake, uptake, and metabolism are available. The strength of this approach is that it demonstrates unequivocally that exposure and uptake have occurred. The primary drawbacks of this approach are the lack of specific physiologically based pharmacokinetic models for many pesticides, and that it integrates exposure over all pathways, so it may not provide information on the primary pathways or routes of interest. A recent case study that examined organophosphate exposures in pregnant women living in an agricultural community demonstrates the strengths and limitations of this approach (Castorina et al. 2003). As a consequence, relatively few studies of farm-workers have attempted to reconstruct exposures. Most studies use biological measurements to understand the relative magnitude of different exposure pathways (e.g., inhalation versus ingestion), to identify risk factors for exposure (e.g., nonuse of personal protective equipment), to evaluate the effectiveness of an intervention in reducing exposure, or to classify individuals into groups in epidemiological studies.

In principle, personal and biological measurements are complementary methods. Because they are based on direct measurements in the exposed population, they are the preferred approaches for pesticide exposure assessment in farmworkers. In practice, however, personal measurements are infrequently used because of resource constraints (e.g., time, personnel, and money), and important data are not available for specific situations and populations of interest. Consequently, the most common approach is scenario-based exposure assessment, which entails the construction of a plausible set of assumptions (i.e., a scenario) that describes quantitatively how contact occurs between people and pesticides. This approach requires the use of available measurements in combination with inferences and professional judgment.

A typical scenario-based approach estimates pesticide exposure by merging the following information: *a*) concentration of the chemical in the carrier medium, estimated by using monitoring, exposure models, or assumptions; and *b*) the individual’s contact time with the carrier medium, estimated by using existing geographic and time-activity or other exposure factors, or by making reasonable assumptions about activity patterns, source proximity, and other relevant factors. Doses are then estimated by using knowledge and assumptions about relevant pharmacokinetic processes. Variations of the scenario-based approach include both *a*)“microenvironmental” methods, which combine measurements in important microenvironments (e.g., inside the residence and outdoors in the community) with data on time-activity patterns (Freeman et al. 2001; Klepeis et al. 2001; Reed et al. 1999; Zartarian et al. 1995), and *b*) pathway-exposure factor methods, which combine measurements in important environmental media (e.g., air, water, food, soil, and dust) with exposure factors (e.g., volume of air breathed or water consumed per day, body weight, and skin surface area) from relevant sources [U.S. Environmental Protection Agency (U.S. EPA) 1997, 2002]. Many of these exposure factor data sets were not collected in farmworkers, and they may not represent variability in behavior or activities for this group (Quandt et al. 2006).

The primary advantage of scenario-based approaches is that they enable assessors to estimate pesticide exposure and dose in cases where data are limited or lacking. The uncertainty introduced by the need to make assumptions and inferences in the face of inadequate or inappropriate information is also their major disadvantage. Scenario-based assessments typically do not include a complete description of the exposure and dose distribution for farm-worker populations. As a result, these estimates may provide only point estimates of specific locations on the population distribution of exposures. Despite their limitations, scenario-based approaches remain the only viable method for estimating pesticide exposure and dose in the absence of personal measurements.

## Sampling Methods

Pesticides may be present in soil, air, water, dust, or food in or on surfaces around the home. To date, a majority of residential exposure studies have collected house dust or surface residue samples to evaluate exposure to pesticides in the home. Past emphasis on house dust and surface residue samples can be explained, at least in part, because these samples are thought (perhaps erroneously) to mimic personal exposure measurements. Also, they are relatively easy to collect compared to air, food, and water samples (Alavanja et al. 2004; Bradman et al. 1994, 1997; Colt et al. 1998; Fenske 1997; Fenske et al. 2002; Moate et al. 2002; Quandt et al. 2004a; Simcox et al. 1995). Because the potential for health effects from pesticides may result from exposure from all routes, the following sections discuss sampling of all environmental media.

### Surface sampling

Surface residue sampling methods are intended to provide some measure of concentration (e.g., micrograms per square centimeter) that can be related to human exposure through a scenario-based assessment. For example, a moist wipe sample from a hard floor surface measures residue that can potentially transfer to hands, which can then be ingested via a hand-to-mouth process in children. Desirable characteristics for surface residue sampling techniques are that they can be standardized so they give reproducible results across studies.

Available methods for surface sampling indoors include the following: *a*) deposition pad samples; *b*) wipe sampling techniques, used on relatively smooth surfaces such as floors, counter-tops, and window sills; and *c*) vacuum techniques, which have been used to collect house dust samples from both hard floor surfaces and carpets. Pesticide deposition sampling is performed postapplication using aluminum foil (Fenske et al. 1991), gauze (Ross et al. 1991), or cotton cloth pads (Byrne et al. 1998; Krieger et al. 1997) as the collection medium. The choice of method used to remove pesticide residues from surfaces can have a significant effect on estimated exposures. For example, investigators who used deposition pads wiped on surfaces and toys subsequently extracted with isooctane report either nondetectable or lower chlorpyrifos exposures (Byrne et al. 1998) compared to investigators who used hexane to remove pesticides directly from surfaces and toys (Gurunathan et al. 1998). Use of organic solvents directly on a surface apparently results in more complete removal of chlorpyrifos residues, but it may overestimate doses obtained in dermal contact or hand-to-mouth activities, where the solvent would be saliva, sweat, or the sebum layer on the skin (Edwards and Lioy 1999).

A number of vacuum sampling systems have been developed to collect house dust samples from carpets, rugs, and bare floors. A specialized high-volume vacuum sampler was developed specifically to obtain samples of semivolatile pesticides in house dust [American Society for Testing and Materials Standards (ASTM) 1993]. It has been used in several field studies to collect carpet and smooth surface samples (Bradman et al. 1997; Lewis et al. 1994; Nishioka et al. 1996; Simcox et al. 1995). The ASTM method obtains similar median but higher upperbound dust pesticide concentrations compared to dust samples from home vacuum cleaner bags, possibly because of a higher collection efficiency compared to commercially available vacuums typically used in residences (Colt et al. 1998).

Three techniques have been developed to measure dislodgeable residues on indoor and outdoor surfaces and to characterize transfer from one location to another. Two of these methods have used polyurethane foam (PUF) as a collection medium (Lewis et al. 1994; Ross et al. 1991). The method of Lewis et al. is designed to simulate the force of a child crawling on a surface. Using this method, investigators estimated 2,4-D and dicamba track-in rates onto carpets after outdoor turf applications (Nishioka et al. 1996). Transfer was estimated to be 3% of dislodgeable residues, and total transfer was estimated to be 0.1 to 0.2% of the total turf application. A “drag sled” method has also been developed that uses a 100-cm^2^ patch of denim affixed to the bottom of a sledlike device, whose weight approximates the force exerted by a 10-kg child on a surface (Byrne et al. 1998; Vaccaro et al. 1996). Similar methods have been developed to assess the dislodgeable residue from treated lawns (Fuller et al. 2001; Klonne et al. 2001; Rosenheck et al. 2001). In theory, the PUF and drag sled methods should give similar results, but no studies have directly compared them.

Use of surface or dust samples poses limitations because such samples do not represent all sources of pesticide exposure, and no current studies link concentrations in dust to specific health effects; however, there are ongoing efforts in this direction. Furthermore, dust samples provide no information regarding food-related exposures and exposures during direct pesticide applications. Dust samples do provide a picture of what is present in the home, the location where a large proportion of the day is spent. Therefore, dust samples should be part of any careful analysis of pesticide exposure in farmworkers.

### Handwipe sampling

Surface dust provides a microenvironmental measure of exposure, while handwipes provide a personal exposure measure. Handwipe methods have been developed that use either isopropanol and gauze wipes (Geno et al. 1996; Lewis et al. 1994; Lu and Fenske 1999) or a 10% isopropanol/distilled water mixture used as a hand rinse to remove pesticides from the skin (Edwards and Lioy 1999; Fenske and Lu 1994). Because of its ease of use the Geno method has been used in several recent studies in farmworker families, for collecting data from hands as well as toys and floors (Curwin et al. 2003; Lewis et al. 2001; Quandt et al. 2004a). The drawback of this method is that it may remove deeply embedded compounds that may not be removable by typical soap and water washing or the hand-to-mouth ingestion scenario. Data from controlled mass-balance experiments suggest that dermal wash methods may significantly underestimate exposure since they typically remove approximately 20–40% of the available compound, with the remaining amounts likely absorbed through the skin (Fenske and Lu 1994). Only one study has compared pesticide residue levels measured by wipe, roller, and handwipe or press methods. Indoor chlorpyrifos levels after broadcast application indicate that wipe and PUF roller measurements estimate a dermal loading that is 23–36 times greater than estimates based on hand press or drag samples (Lu and Fenske 1999). These data suggest that while measurement methods may be internally consistent and may correctly rank order individuals, comparisons across studies may be difficult because the collection procedures may result in different levels being reported.

### Air sampling

Pesticides can be measured in both indoor and outdoor air in both the gas and particulate phases. The general considerations for sampling indoor or outdoor air concentrations as well as personal air measures are the same and include selection of the type of airflow device, the sample collector and medium, and the sample location. The basic decision regarding type of airflow device is whether to use a high-volume or low- volume sampler. A main advantage of high volume samplers is that they can operate at higher flow rates (> 5 L/min), which reduces the time required to collect a given sample volume. Larger sample volumes require less sensitive analytical methods to achieve meaningful detection limits. However, high-volume samplers are more typically employed in ambient air pollution sampling than in residential exposure monitoring because they are larger, noisier, and less portable than low-volume samplers and often require electrical power to operate. Low-volume samplers generally are portable, battery operated, relatively quiet, and easy to use and are generally used for personal monitoring. Flow rates of 0.5–1.5 L/min are typically recommended for pesticides (Quackenboss et al. 2000). Higher flow rates may be necessary to achieve detection with very short collection times (< 30 min). This might be the case, for example, in trying to measure serial concentrations during and after a fogger application or during and after application of a pressurized aerosol product by or from spray drift from an agricultural application. As with all methods, if the anticipated pesticide concentration is low, the amount of sample collected needs to be increased.

The decision about which sample collector to employ in residential air sampling is primarily influenced by the physical state of the pesticide in air and particle size of the aerosol (Lewis and Gordon 1996). Different collector types can be employed in air sampling, but the primary ones used to sample pesticides are filter cassettes and solid sorbents. Filter cassettes are typically employed if the pesticide of interest exists as an aerosol (solid or liquid), and solid sorbents are typically used for vapors. These collectors can also be used in series if there is a chance for the pesticide to be present in both phases, or to minimize sample loss caused by volatilization off the filter cassette, which can occur during extended sampling periods. Most pesticides are present as aerosols during and for short periods following application and then exist as vapors or bound to dust particles. Resuspension through vacuuming, dusting, or other mechanical means can also contribute to aerosols being present postapplication.

### Food and water sampling

Dietary exposure to pesticides can occur via both food and water, and no studies have systematically evaluated this specific pathway for contribution to overall farmworker exposure. In the studies that have been done for the general population, evaluation of dietary exposure frequently involves mathematical modeling of exposure based on food consumption patterns and information on the levels of pesticides in those foods (Clayton et al. 2003; MacIntosh et al. 1996, 1999b, 2001). The accuracy of the estimates depends then on the confidence one has that the measurements of the residue levels in foods are realistic and reflect the actual pesticide residue levels. A more intensive assessment of pesticides in food comes from the “duplicate plate” method, wherein exact duplicates of a person’s daily meals and snacks are prepared concurrently with their consumption and then measured for pesticide residue levels (Clayton et al. 2003). Collection, storage and analysis of food samples as well as recruitment of highly motivated participants can be limitations to conducting these studies in any population, and may be especially difficult in farmworkers because of education, language, and proper incentives for participation in research. In some communities, primarily those relying on well water for drinking water, pesticide exposure may occur through use of this water for drinking, cooking or bathing. In most research conducted to date, exposure to pesticides via drinking water is low, largely because pesticides in both wells and public water systems have been diluted over time by the large volume of ground or surface water (MacIntosh et al. 1999a). Although water samples are easy to collect at the tap, large volumes may be necessary since the concentrations are generally at or below analytical detection limits. Farmworker exposures from drinking water are likely to be small relative to occupational, food, or residential exposure sources unless the drinking water source is highly contaminated or water consumption is very high.

## Design and Conduct of Exposure Studies

**Figure 2:**
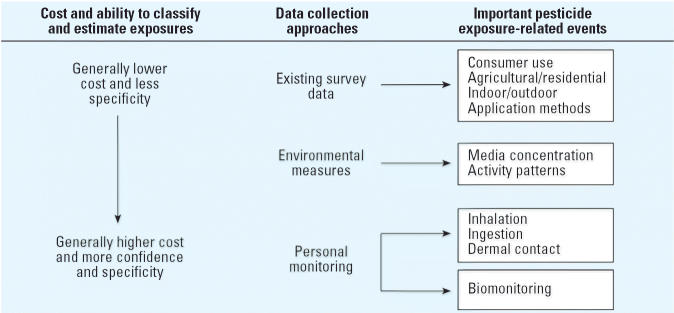
Hierarchy of exposure information.

Exposure assessment focuses on identifying the contaminant exposures experienced by individuals as they go about their daily activities. Table 1 outlines four major exposure assessment strategies with regard to the research question, the study design, the exposure measurement scenario, and the outcome measures and limitations of each type of study. These studies differ in complexity and specificity of information required. Figure 2 illustrates the possible levels of detail in exposure information. Issues for selection of environmental media and analysis of environmental samples are discussed below.

Design of pesticide exposure assessment studies needs to consider both the analyte of interest and the exposure pathway of interest. The first step is to determine the pesticides that are being used in the region, often not an easy question to address, and the next step is to identify whether appropriate collection and analysis methods exist. Selection of analytes determines the media of interest, the sampling approach, other potential analytes, and the cost, feasibility, and logistics. Investigators may choose to focus on both agricultural and residential pesticides to capture a complete picture of total pesticide exposure. The physical and chemical properties of pesticides can be used to identify where and when to find a pesticide. Sample location is also an important consideration in collecting residential samples. The main objective should be to collect samples from a location(s) that is representative of where farmworkers spend time. These locations could include vehicles or changing areas, but they should also encompass living rooms, kitchens, bedrooms, or other rooms where farmworkers spend time.

Exposure assessment studies can use either random samples from a defined population, such as farmworkers in a specific geographic area, or a convenience sample of available workers. Convenience sampling affords selection of individuals for monitoring based on criteria other than statistical representability. Advantages of the convenience sample are ease of selection and likely increased compliance with measurement protocols because of higher personal motivation, but results may not be representative of the larger population or, more importantly, of the overall distribution of exposures. Intervention studies often start with convenience samples to assess whether the intervention works among highly motivated individuals before approaching a larger population. A representative sample is obtained using a random sampling approach to select individuals. While a representative sample is more easily generalizable to a larger population, the downside of this approach is that it may result in reduced compliance and potentially, loss to followup. Also, less motivated participants often reduce the reliability of such investigations.

The temporal pattern of exposure is a key design issue for all exposure assessment strategies. The timing of sample collection needs to consider transport and dispersion mechanisms that are important in the scenario being studied, and to merge these factors with design considerations and data concerning acute or chronic situations or the decay from acute to low-level residual exposures. For example, aerial spray applications may result in more rapid transport of pesticides from farm field to residence than in-ground applications to row-crops. Thus, for aerial spray applications, air monitoring in homes near fields may need to coincide with the initiation of application. However, if transport of residues on contaminated clothing is the greater concern, sampling that coincides with the entry of that clothing into the home may be used to ascertain the relative contributions of sources. Similarly, the collection of dust residues (either with wipe techniques or by vacuum sampling) may take up to a week for dispersion and equilibration to occur within the home, but this timing needs to be weighed against individual or cultural practices regarding the frequency of residential cleaning. Another important temporal consideration is that because new pesticides are introduced to the market constantly, methods need to be flexible to accommodate market changes in pesticide use.

### Surveillance studies

Surveillance studies address the following questions: *a*) are pesticides present? *b*) in what media are they present? *c*) at what levels? and *d*) what factors predict pesticide presence? In these studies researchers may collect and analyze samples such as air, water, dust, soil, and dislodgeable surface residues to determine the pesticide(s) of interest. Surveillance studies are important steps to undertake, as it is through these studies that we understand the situations and scenarios where pesticide exposure might occur. For example, until lawn pesticides were measured in house dust (Nishioka et al. 1999, 2001), we did not have the foundation for hypothesizing the transport mechanisms and activities whereby children could be exposed to these pesticides while indoors. Surveillance studies can also serve an important role as the preliminary step to larger more comprehensive personal exposure characterization studies. The surveillance studies may help to establish the range of levels that can be encountered so that power calculations can be performed or so that analytical techniques and detection limit requirements can be set. These studies may also be used to assess whether and to what extent other factors such as physiochemical properties or agricultural use affect the transport from occupational to residential setting. As such, a comprehensive exposure assessment study may not always be required if there are existing factors that link pesticide concentrations together with activity patterns and exposure factors to potential exposure.

Surveillance studies frequently rely on convenience samples to assess potential exposure. Limitations associated with convenience samples can be minimized by screening of initial study respondents and selecting for variability in factors that might affect exposure measures, such as proximity to treated fields, job classification, method of pesticide application, personal hygiene, and protective practices. Postsampling questionnaires may uncover additional factors that broaden the applicability of the results. These questionnaires may cover factors related to lifestyle (i.e., showering after work and laundering of work clothing), parent–child interactions (whether children are allowed in mixing, loading, and application areas), and familial activity factors (such as whether pets are allowed indoors and frequency of vacuuming and cleaning). Questionnaires and other metrics that predict exposure factors are discussed in the article by Quandt et al. (2006) in this mini-monograph.

While surveillance studies do not have the same requirements as an exposure characterization study for tightly linking sampling method, sampling location, and sample timing to an exposure metric, these features are still important design requirements. Careful consideration needs to be given to the population, to the timing and frequency of sample collection with respect to pesticide use, and to the issue of spatial distribution within a sampled medium.

### Exposure characterization

Exposure characterization determines the presence of a pesticide or its metabolites in environmental media. Exposure characterization builds on exposure surveillance designs to further assess the temporal and spatial patterns of exposure in the population. The temporal and spatial patterns of exposure are assessed through the collection of multiple samples to describe the behavior of pesticides in that environment. This may focus on just one medium (e.g., air) or multiple media to evaluate total exposure. Collection of samples from all relevant environmental media allows the assessment of the relative contribution of each source or pathway to an individual’s total pesticide exposure. Exposure characterization may rely on historic data such as pesticide application records and wind direction and use mathematical models to estimate exposure levels over time or may involve an intensive field effort to collect samples.

Before the development of a protocol to assess the distribution of human exposure, one must make several decisions. First, it is necessary to define the population for which the exposure distribution is to be measured. An additional consideration is temporal variation. Certain exposures may be relatively constant over long periods of time, whereas others may be episodic, random, or vary seasonally. Cross-sectional monitoring may be sufficient to ascertain the exposure distribution if exposures are constant over time. If exposures vary over time, especially in cases where such variation cannot be easily modeled, it may be necessary to monitor longitudinally. In such a design, a group of farmworkers may be monitored repeatedly, with each monitoring period being equivalent to one cross-sectional study. A cross-sectional study can also encompass a larger initial population, thereby affording better assessment of the population distribution; however, temporal variability in exposure within individuals in the population as well as within the overall population of farmworkers cannot be addressed in such a study. Longitudinal investigations can assess such variability, but they are more limited in sample size because costs typically increase more with the number of individual measurements rather than with the number of individuals monitored. Attrition limits the utility of longitudinal studies for determining overall exposure distributions, particularly among transient populations such as migrant farmworkers.

One strategy to assess both population and individual variation is the use of nested designs. In such designs, a large sample is chosen initially, and monitoring is accomplished via simple and inexpensive instrumentation, often using only questionnaires. A subset of this group is then selected for more intensive monitoring. In principle, many levels of such nesting are possible while maintaining a probabilistic approach. Nested designs are commonly employed in epidemiologic investigations; for example, the Agricultural Health Study (Alavanja et al. 1996), a large case–control study of farmers and their families, has conducted intensive exposure monitoring on a subset of the cohort to help validate the exposure assumptions derived from questionnaires (Agricultural Health Study 2006). The larger components gather information about many individuals, and the more detailed data collection affords placement of individuals on the distributional scale.

### Intervention studies

Intervention studies use exposure assessment to evaluate whether the intervention was successful at reducing pesticide exposure levels in homes. These studies compare the levels among treatment groups and thus may be appropriate for pooling samples within a house or across houses. Intervention studies, like other exposure studies, may also involve reporting results back to participants, so these studies benefit from the availability of comparable data to share with participants (Quandt et al. 2004b). Understanding the temporal and spatial variability is key to the design of intervention studies so that the effect measured is associated with the intervention and not just the sampling variability. This requires careful selection of measurement, assessment, and statistical techniques that will be used to evaluate the effectiveness of the intervention.

### Epidemiologic studies

Epidemiologic studies are conducted to specifically assess whether pesticides are associated with health effects in populations. Since most adverse health outcomes are rare, investigators need to balance the quality of the exposure data with the sample size required to detect an outcome in an epidemiologic study. Improvement of exposure sensitivity (e.g., individual level exposures versus categorization) may reduce sample size sufficiently to afford a less expensive study despite the increased exposure monitoring costs. If this is not possible, then the typical approach is to obtain a larger populations than a typical exposure assessment study. As a result, the exposure assessment strategies may be less quantitative than those used in surveillance or exposure characterization studies. Exposure may be classified as ever/never or in quantiles of exposure and thus precision in the absolute concentration may be less important than an individual’s relative location in the exposure distribution. Because of the need to assess exposure on a large number of individuals, relatively inexpensive exposure measures are often employed, with nested studies conducted to validate the exposure assumptions (Alavanja et al. 2004; Kromhout and Heederik 2005). Depending on the health outcome, the time period for the exposure of interest is likely to have already occurred, resulting in the need to obtain retrospective measurements of exposure (Alavanja et al. 2004). Understanding the temporal and long-term variability of pesticides in environmental and biological media will allow further application of these measures in epidemiologic studies.

## Comparing Across Studies

Comparison across studies can be challenging because each study has different objectives, but this process can be facilitated if common factors are recognized and standardized during the study design phase (Table 2). Specific key factors include active ingredient, geographic region, calendar year, and season of sampling. Comparisons are simpler if the same pesticide and the same medium are sampled, but even when considering a widely used compound and collection medium such as chlorpyrifos in house dust, differences in the sampling and analysis protocols may limit the validity comparisons. Dust sample results need to consider the method of collection (wipe or vacuum), the size fraction analyzed, and the sampling location within the household. For wipe samples, the solvent used in the wipe sample and the method of extraction may also influence the comparability of results. Detection limits for the pesticide of interest and the statistical reporting of results may also affect the reported distribution. If nondetects are included as zero values, while other investigators exclude the nondetected values, the means and the distributions in the populations will differ greatly.

Given these many possible sources of variability in exposure between studies, it is important that priorities are set to guide future studies and move the science forward. Although all properly conducted studies are internally valid, there are still things that researchers can do that will allow easier comparisons among studies. First, all studies should include information on response rates, representativeness of the sampling frame (calendar year, region, population sampled, etc.), valid quality assurance measures, and adequate statistical power to address the question(s) of interest. New studies need to use documented methods or include the following key information on all aspects of the exposure assessment: sample collection, chemical, and statistical analysis and interpretation of what the sample represents. Given the importance of surface sampling to estimate exposure and health effects in farmworkers, additional research is needed to aid interpretation of dust and dust sampling wipe results. Specifically, better estimates of pesticide transfer rates from floors to hands and dermal absorption rates are required. For example, a study of direct head-to-head comparisons of a number of sampling methods, both in laboratory and field settings, would provide the opportunity to know when data from the methods were comparable and which methods performed better under different conditions. The overall goal needs to be standard exposure metrics that permit valid comparisons of dose–response results between studies or meta-analyses across studies. One additional key issue in studies of farmworker families is presentation of results to the farmworker community and other lay audiences in terms they can understand, even if scientists are uncertain of the health implications of the measured exposure (Quandt et al. 2004b).

In summary, a variety of methods exist to collect samples to assess potential exposure to pesticides from environmental media. To date, most of the research has focused on dust sampling, but there has been little standardization with regard to the methods used. For example, the results from vacuum surface sampling are generally reported in mass concentration (micrograms of pesticide per kilogram of dust), while the results from wipe and surface samples are typically reported as surface loading (micrograms of pesticide per square centimeter of floor). While surface loading is most likely more closely related to exposure and health effects than dust concentration, little research has been conducted on this topic. In our judgment, results obtained via surface loading are not likely to be correlated strongly with those obtained via dust concentration. Therefore, results obtained by these two different methods are difficult to interpret, and a comparison of the data may be limited to rank ordering. In addition, vacuum measurement methods with uncharacterized particle size collection characteristics may be difficult to interpret or to compare to more rigorous methods that collect a known size fraction, which can be related to an specific exposure scenario. Results from wipe samples need to be considered carefully, because the method of collection, such as a handwipe versus a solvent wash, may affect the amount of pesticide removed from a hand. A systematic framework is needed to develop consistent and reproducible methods to be used in future pesticide exposure studies in farmworker families. We have outlined the first steps toward this framework, but future research will help to shape its ultimate form.

## Implications for Future Studies

To address pesticide-related health effects in farmworkers, the determination of the contribution of these residential exposures is critical for characterizing total exposure, estimating potential health risks, implementing effective interventions, providing benchmarks for assessing environmental justice, and conducting epidemiological studies. Currently, research has focused on surveillance studies and on identifying pesticides present in dust and on surfaces in farmworker homes. Measuring dust levels of pesticides is important, because dust appears to be one of the most important sources of pesticide exposure, given the small contribution of water and air. Future work should focus both on better understanding of how the dust and hand-wipe collection methods compare and on collecting repeated measurements to ascertain the temporal and spatial variation in pesticide levels in farmworker homes. One area that is poorly studied among farmworkers, as well as the general population, is the level of pesticides in food and the potential exposures associated with food consumption. Because direct measurement of pesticides in food is time consuming and difficult, modeling strategies that incorporate food consumption habits should be employed to assess this potentially important source of pesticide exposure. As the field matures, and the surveillance studies are applied to health effects studies, understanding the exposure assessment methodologies and their comparability will be critical to conducting risk assessments and to determining mitigation strategies.

## Figures and Tables

**Table 1:** Research questions and study designs that employ environmental exposure measurements.

Hypothesis/question	Study design	Exposure measurement/scenario	Outcome/limitations
Are pesticides present in the residence?	Surveillance	Measurement: pesticides in air, water, soil, house dust, surfaces	Distribution of residential concentrations, predictive factors No direct link to exposure or health effects
What is the distribution of human exposures?	Exposure characterization	Media measurements, biomonitoring, and exposure factor characterization one or more times	Distribution of individual potential doses, estimation of high-end exposures, relatively small numbers because of resource limitations
Does an intervention reduce pesticide concentration in a residence?	Intervention	Paired samples before/after intervention	Reduction of environmental concentrations that likely influence exposure
What is the relationship between pesticide exposure and a health effect?	Epidemiology		Outcome: associations between pesticide exposure metric and health effect
	Cross-sectional	Concurrent potential exposure and outcome measurement	Limitation: potential problems with temporal sequence of exposure/effect
	Retrospective	Reconstructive analyses	Limitation: assumptions about past probability of contact and concentrations in the environment or body
	Prospective	Exposure measurement before disease; longitudinal measurements	Time varying exposures; cost, critical time period of exposure

**Table 2:** Factors to be considered when collecting environmental samples for health studies in farm-workers.

Factors that can be standardized
Media sampled
Dust, soil, water, food, handwipe
Collection methods
Wipe
Vacuum
Size fraction analyzed (dust, soil, air)
Sampling location
For example, child’s bedroom
Analytical methods
Detection limits
Volume of sample collected
Site-specific factors
Analytes of interest
Geographic region
Crops raised, pests of concern
Calendar year
